# A French nationwide study compared various conditions and healthcare use of individuals < 65 years with a Down’s syndrome to those without

**DOI:** 10.1038/s41598-023-49102-4

**Published:** 2023-12-10

**Authors:** Philippe Tuppin, Pauline Barthelemy, Gonzague Debeugny, Antoine Rachas

**Affiliations:** Direction de la Stratégie, des Etudes et des Statistiques, Caisse Nationale de L’Assurance Maladie, 26‑50, Avenue du Professeur Andre Lemierre, 75986 Paris Cedex 20, France

**Keywords:** Genetics, Cardiology, Diseases, Endocrinology, Gastroenterology, Health care, Neurology, Oncology, Risk factors, Signs and symptoms, Urology

## Abstract

Few regular national clinical data are available for individuals with Down’s syndrome (IDS) bearing in mind that they are subject to countries variations in medical termination of pregnancy and screening. Individuals < 65 in 2019 were selected in view of the low number of older IDS. Thus, 98% of 52.4 million people with correct data were included from the national health data system. IDS (35,342) were identified on the basis of the International Classification of Diseases 10th revision code (Q90). Risk ratios (RR) were calculated to compare the frequencies in 2019 between IDS and individual without Down’s syndrome (IWDS) of use of health care. The prevalence of IDS was 0.07% (48% women), comorbidities were more frequent, especially in younger patients (24% < 1 year had another comorbidity, RR = 20), as was the percentage of deaths (4.6%, RR = 10). Overall, tumours were less frequent in IDS compared with IWDS (1.2%, RR = 0.7) except for certain leukaemias and testicular tumours (0.3%, RR = 4). Cardiac malformations (5.2%, RR = 52), dementia (1.2%, RR = 29), mental retardation (5%, RR = 21) and epilepsy (4%, RR = 9) were also more frequent in IDS. The most frequent hospital diagnoses for IDS were: aspiration pneumonia (0.7%, RR = 89), respiratory failure (0.4%, RR = 17), sleep apnoea (1.1%, RR = 8), cryptorchidism (0.3%, RR = 5.9), protein-energy malnutrition (0.1%, RR = 7), type 1 diabetes (0.2%, RR = 2.8) and hypothyroidism (0.1%, RR = 72). IDS were more likely to use emergency services (9%, RR = 2.4), short hospital stay (24%, RR = 1.6) or hospitalisation at home (0.6%, RR = 6). They consulted certain specialists two to three times more frequently than IWDS, for example cardiologists (17%, RR = 2.6). This study is the first detailed national study comparing IDS and non-IDS by age group. These results could help to optimize prenatal healthcare, medical and social support.

## Introduction

Down’s syndrome (DS) is the most prevalent chromosomal abnormality (a third copy of chromosome 21 in 96% of cases, followed by translocation or mosaicism). DS causes intellectual disability of variable severity and is characterized by diverse additional clinical manifestations^1^. No specific risk factors have been identified, other than advance maternal age at delivery and a previous pregnancy in which the foetus had DS^1,2^. With advances in screening and diagnosis, DS can now be confirmed at the antenatal and neonatal periods, with cases of mosaic DS being diagnosed at a later stage^2^. Cell-free prenatal screening and parallel sequencing of maternal plasma cell-free DNA have also reduced the use of invasive testing (i.e. amniocentesis or chorionic villus sampling)^3^.

The incidence and prevalence of livebirths of individuals with DS (IDS) have varied over time and between countries, depending on the demographic characteristics of the population (older mothers, mixing of different populations through migration, family size, postponement of first pregnancy, cultural factors, etc.) and on DS screening, diagnosis, potentially treatable cardiac or gastrointestinal defects and policies concerning elective abortion, the dissemination of information, access, consent and financial support and restrictions of elective abortion according to the conditions detected in the foetus with DS^2–5^. In the USA, an increase in the prevalence of DS was observed due to the migration of Hispanic and Asian individuals during their fertile years, but this prevalence has remained stable in the face of the decrease in the non-Hispanic white population. Elective abortions have a limited impact, decreasing the proportion of livebirths affected by DS by 19% (prevalence of 0.067% in 2010)^6,7^. Estimates of the number of IDS alive in 2015 based on regional birth registries and modelling techniques have been published for many European countries with diverse rates of elective abortion (0–83%)^8^. In France, the population of IDS was estimated at 35,684 people, with a prevalence of DS over all age groups of 0.056% and of 0.096% if elective abortions were also taken into account (41%)^8^.

The life expectancy of people with DS has greatly increased, reaching 60 years, with improvements in knowledge, medical care specialization, adaptation and research^1,9,10^. Childhood survival has improved, but excess mortality persists, due to the severity of congenital heart defects^11^. Leukemia/lymphoma, pneumonia and low birthweight are also associated with earlier death^12^. In adults with DS, the leading causes of death are congenital heart abnormalities and respiratory conditions^13–15^. There is a need to improve the treatment of aging-related disorders or diseases with an earlier onset in the context of DS, such as Alzheimer’s disease, with an adaptation of transitions in healthcare^6–21^.

The prevalence of common disease conditions and causes of death have mostly been reported for specific conditions and in a few recent large cohorts of IDS or population studies not taking healthcare use into account^14,20–22^. Hospital diagnoses over the individual’s lifespan are not detailed for chronic and acute diagnoses^22–24^. Comparisons of the annual use of various healthcare is possible with the SNDS (the French national health database), which provides information about health conditions and healthcare reimbursements almost the entire French population; given that health insurance coverage is universal in France^25^.

We performed the first national observational study comparing a large population of IDS to individuals without Down’s syndrome (IWDS) over 2019. This study focused on DS prevalence, social and demographic characteristics, comorbid conditions and mortality. The annual frequencies of medical visits by specialty, emergency department visits and hospital admissions were also determined.

## Materials and methods

### Evolution of French data on children and mothers characteristics and DS screening

The number of children born in France was around 800,000 per year at the end of the 1970s, falling to 741,000 in 1994. There was then a steady increase to a peak of 830,000 in 2012, followed by another rapid fall (753,000 in 2019 and 720,000 in 2022) (INSEE, National Institute for Statistics and Economic Studies)^26^. In 2022, women gave birth to their first child at a mean age of 31.0 years, four and a half years later than in 1974. In 2019, 42,800 babies were born to mothers aged 40 or over (5.7% of births were so-called "late births"), 25% of newborns were the first children of mothers aged 35–44 years and 36% were the first children of mothers aged 45–50. For every 100 women born in France, eight children were born to mothers between the ages of 40 and 50 years, whereas the corresponding figure was 19 children for women born elsewhere. Women in managerial or higher intellectual occupations had the highest rates of late deliveries among women who had already worked: 11 children per 100 women between the ages of 40 and 50 years.

It has been possible to perform cytogenetic analysis on cultured amniocytes since the 1970s. This has made prenatal diagnosis feasible in pregnant women aged 38 years and over and this procedure is reimbursed in France. The first recommendations for population-based screening concerned the use of serum markers in the second trimester of pregnancy^27^. The possibility of earlier prenatal diagnosis based on chorionic villus sampling in the 1980s met the need for screening in the first trimester.

### Data source

The SNDS collects individual information from the various insurance schemes, the types of healthcare used and the amounts reimbursed^25^. It does not record diagnoses made in non-hospital settings or the results of clinical examinations and investigations. However, it does include information about long-term disease (LTD) status for a specific condition, which justifies 100% reimbursement of specific healthcare expenditures for at least five years, if requested by the patient’s general practitioner (GP). The list of LTDs is published by decree. A pseudonymized identification number is used to link above information with data from the national hospital discharge database concerning stays in public and private hospitals: short-stay hospitalization (SSH), hospitalization at home, or hospitalization in psychiatric hospitals and rehabilitation facilities. LTD and hospital diagnosis are coded using the International Classification of Diseases 10th revision (ICD-10). Deaths are also collected in the SNDS.

For comorbidities, we also used data from “Healthcare Expenditures and Conditions Mapping”, a tool based on the SNDS developed for the analysis and monitoring of disease burden through evaluations of healthcare use^28^. This tool can be used to identify 58 diseases or chapter of diseases through the use of medical algorithms based on the reasons for hospitalization, long-term disease diagnoses and the reimbursement of specific treatments for certain diseases, for a given year and a period up to four years preceding that year. For tumors, exclusive algorithms identified different states of the same disease related to active treatment or acute care or to long-term follow-up and chronicity or under surveillance.

### Study population

This large cross-sectional observational study focused on individuals with national health insurance coverage receiving at least one healthcare reimbursement in 2019 (98.5% of the population residing in France: 67.25 million on January 1, 2019 according to the INSEE and 52.3 million aged < 65 years)^26^ for estimations of overall prevalence. We subsequently focused solely on individuals under the age of 65 years, given the very low frequency of IDS over this age relative to the number of IWDS. Individuals who died in 2019 were not excluded from the year of follow-up. DS was identified by the presence of the ICD-10 code for DS (Q90) in the information about LTD status or hospital diagnoses from 2015 to 2019 and to 2020 for people born in 2019.

### Variables analysed

It involved: age class (as of January 1, 2020), sex, residence in a French overseas territory (FOT, including the French West Indies, French Guiana, and Réunion Island), date of death, comorbid conditions (categories and frequent specific detailed diseases from Healthcare Expenditures and Conditions Mapping, together with any LTDs with a frequency ≥ 0.1% for the DS group), diagnoses during hospital stays (by chapter, and detailed diagnoses with a frequency ≥ 0.1% associated with at least one hospital stay for the DS group, to identify admissions for acute disease or requiring a hospital procedure, for acute and chronic conditions) and hospital care and medical visits.

C2S (*Complémentaire santé solidaire*), a solidarity-based supplementary health insurance, is a renewable benefit granted for one year to people with a limited resources and a stable and regular residence in France. The household covered by C2S includes the applicant, the applicants spouse or partner and any children. In 2020, the annual income limit in mainland France for C2S eligibility was between €9,032 and €12,193 for a single person. This limit is below the poverty threshold, defined as 60% of the median income, which was €13,224 in France in 2019.

### Analyses

The prevalence of DS was estimated in 2019, globally and for the population of individuals under the age of 65 years. For this second population, we compared individual characteristics, healthcare use and comorbid conditions between IDS and NIDS, on the basis of rate ratios (RR). Finally, we considered the principal diagnosis during hospitalization for individuals with at least one SSH.

We also compared primary healthcare consultation and hospital use in 2019. The median number and interquartile range (IQR) were calculated for the number of medical visits, for individuals with at least one such visit, to assess differences in the intensity of healthcare use between IDS and IWDS. We also analyzed the male and female populations separately, to check for sex-specific conditions. Given the almost exhaustive nature of the population and the large sample size, we report crude RR without 95% confidence intervals and *p*-values^29^.

SAS software (version 7.13, SAS Institute Inc, Cary, NC, USA) was used for statistical analysis.

### Ethical approval

A specific ethics committee approval was not required for this study. The French national health insurance (CNAM) in charge of the SNDS (système national des données de santé) has permanent access to the pseudonymized reimbursement data in application of the provisions of articles R. 1461‐12 et seq. of the French Public Health Code with rules and criteria similar to Helsinki declaration. The CNAM has permanent full access to the SNDS by decree (Décret n° 2016–1871 du 26 décembre 2016 relatif au traitement de données à caractère personnel dénommé « système national des données de santé ») The CNAM has authorization to perform studies based on SNDS data from the CNIL (National independent Commission for Computing and Freedom, the French data protection agency as sensitive information). All methods were carried out in accordance with relevant guidelines and regulations in accordance with the Declaration of Helsinki.

## Results

### Population characteristics

**Figure 1:**
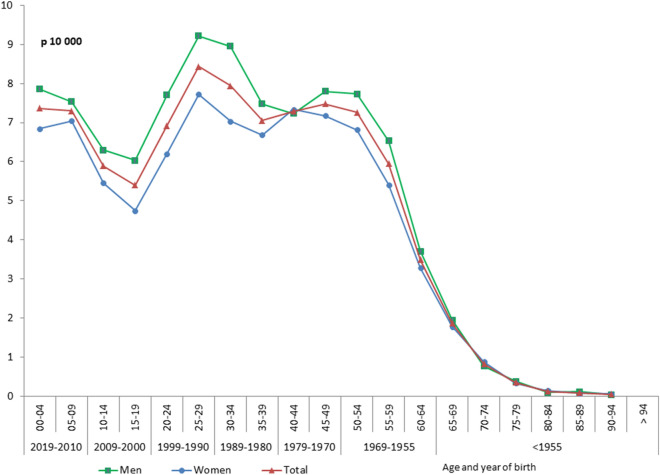
Prevalence of Down’s syndrome in France in 2019 by sex, age and year of birth (N = 36,464).

In 2019, the prevalence of DS was 0.055% in individuals of all ages (Fig. 1) and 0.068% when IDS over the age of 65 years were excluded (3% of the 36,464 IDS). For IDS under the age of 10 years (born in 2009–2019) the prevalence was relatively uniform, at about 0.07%. IDS prevalence decreased to 0.05% for those aged 10–19 years (born in 2000–2009) and increased to 0.08% for those aged up to 30 years (born in 1990–1999). Prevalence then stabilized at about 0.07% until the age of 54 years, after which it decreased rapidly.

Women accounted for 48% of cases of DS (Table 1). A quarter of IDS had at least one other LTD (RR = 2.3). This proportion was uniform over the different age groups, but RR values decreased with age, due to an increase in the frequency of other LTDs with age in IWDS (1 year, RR = 20.1; 50–64 years, RR = 1.3). The proportion of IDS was higher in FOT, particularly for the youngest age group (< 1 year 0.15%, RR = 2.3; 50–64 years 0.05%, RR = 0.8).

**Table 1:** Comparison of sociodemographic characteristics, LTD and death frequencies by age between individuals with and without DS (rate ratio).

Age (years)		Total < 65		< 1		1–4		5–9		10–14		15–19		20–29		30–39		40–49		50–64	
Non DS N (Million)	DS N	52.3 RR	35,342 %	0.7 RR	485 %	3.0 RR	2,230 %	4.0 RR	2,903 %	4.0 RR	2,355 %	3.9 RR	2,104 %	7,5 RR	5,773 %	8.2 RR	6,163 %	8.4 RR	6,211 %	12.7 RR	7,118 %
Women		**0.9**	47.7	**1.0**	48.7	**0.9**	44.6	**1.0**	47.1	**0.9**	45.4	**0.9**	43.7	**0.9**	47.6	**0.9**	48.2	**1.0**	50.6	**0.9**	47.9
Other LTDs (DS excluded)		**2.3**	24.3	**20.1**	23.9	**13.8**	28.4	**7.8**	23.2	**5.4**	19.5	**5.1**	20.7	**4.5**	20.5	**3.1**	21.4	**1.9**	23.7	**1.3**	32.5
Residence in FOT (vs mainland)		**1.2**	0.08	**2.3**	0.15	**1.5**	0.11	**1.6**	0.11	**1.6**	0.09	**1.4**	0.08	**1.5**	0.11	**1.0**	0.08	**0.7**	0.06	**0.8**	0.05
C2S (deprivation)		**1.7**	24.6	**2.2**	42.9	**1.8**	41.4	**1.8**	37.3	**1.8**	34.3	**1.7**	30.3	**1.4**	20.2	**1.3**	17.7	**1.6**	18.9	**2.3**	22.6
Deceased		**10.2**	4.6	**18.9**	4.3	**22.5**	0.9	**16.8**	0.3	**11.7**	0.3	**7.8**	0.4	**6.4**	0.6	**7.7**	1.3	**9.0**	3.7	**12.5**	16.9

*DS* Down’s syndrome, *RR* rate ratio, *LTD* long-term disease, *FOT* French overseas territories, *C2S* solidarity-based supplementary health insurance (low income).

Social precariousness, defined in relation to a threshold for low income (that used for C2S eligibility), was more frequent for IDS over all age ranges (24.6%, RR = 1.7), but particularly for the youngest, whose income was entirely dependent on their family or household (< 1 year, 43%, RR = 2.2).

Overall mortality was higher in IDS under the age of 65 years (4.6%, RR = 10.2) (Table 1). Mortality for IDS under the age of one year was particularly high and considerable excess was noted (4.3%, RR = 18.9) and also for those aged 1–4 years (0.9%, RR = 22.5), or 5–9 years (0.3% RR = 16.8). Mortality subsequently reached a relatively stable plateau, with an RR of between 6 and 12. Mortality then began to increase again in the DS population after the age of 30–39 years, to reach 16.9% between the ages of 50 and 64 years.

### Comorbid conditions

The mapping tool showed that tumors were generally less frequent for IDS than IWDS (1.6%, RR = 0.7), whether these tumors were under surveillance (0.7%, RR = 0.7) or treated (0.9%, RR = 0.8) or the patients were hospitalized at least once in 2019 (0.5%, RR = 0.5) (Tables 2, 4). However (Table 3), myeloid leukemia (0.1%, RR = 4.6) and lymphoid leukemia (0.2%, RR = 6.1) were more common among IDS, particularly for younger. A similar result was obtained for testicular tumors (0.3%, RR = 4.5) (Table 2) and young IDS were also hospitalized more frequently than IWDS for abnormal testicles or cryptorchidism (0.3%, RR = 5.9) (Table 4).

**Table 2:** Comparison of diseases managed, using mapping tools*, by age between individuals with and without DS (rate ratio).

Age (years)		Total < 65		< 1		1–4		5–9		10–14		15–19		20–29		30–39		40–49		50–64	
Non DS *N (Million)*	DS N	52.3 RR	35,342 %	0.7 RR	485 %	3.0 RR	2,230 %	4.0 RR	2,903 %	4.0 RR	2,355 %	3.9 RR	2,104 %	7,5 RR	5,773 %	8.2 RR	6,163 %	8.4 RR	6,211 %	12.7 RR	7,118 %
Tumor		**0.7**	*1.6*	**52.5**	*1.0*	**20.4**	*1.6*	**10.1**	*1.3*	**8.1**	*1.1*	**4.5**	*0.9*	**2.2**	*0.9*	**1.0**	*1.1*	**0.8**	*1.9*	**0.5**	*2.8*
Under surveillance		**0.8**	*0.9*	**219.0**	*0.6*	**40.4**	*0.6*	**10.6**	*0.6*	**9.2**	*0.7*	**6.3**	*0.6*	**2.9**	*0.6*	**1.3**	*0.7*	**0.8**	*1.0*	**0.5**	*1.5*
Active		**0.7**	*0.7*	**24.5**	*0.4*	**15.7**	*1.0*	**9.6**	*0.7*	**6.7**	*0.4*	**2.6**	*0.2*	**1.5**	*0.3*	**0.7**	*0.4*	**0.7**	*0.8*	**0.5**	*1.4*
Haemophilia and haemostasis disorders		**1.8**	*0.1*	**0.0**	*0.0*	**2.1**	*0.0*	**6.0**	*0.2*	**1.1**	*0.0*	**3.2**	*0.1*	**2.1**	*0.1*	**1.8**	*0.1*	**1.8**	*0.2*	**1.2**	*0.1*
Diabetes		**1.1**	*3.2*	**0.0**	*0.0*	**11.4**	*0.4*	**4.5**	*0.6*	**4.0**	*1.0*	**3.6**	*1.3*	**4.4**	*2.3*	**2.7**	*2.9*	**1.7**	*4.7*	**0.7**	*6.1*
Psychiatric disorders		**2.1**	*7.9*	**5.4**	*0.6*	**4.3**	*2.1*	**2.1**	*3.1*	**1.9**	*3.7*	**1.8**	*4.6*	**2.1**	*5.4*	**2.2**	*7.4*	**1.9**	*9.8*	**2.4**	*15.4*
Psychotic conditions		**1.4**	*1.0*	**0.0**	*0.0*	**0.0**	*0.0*	**0.0**	*0.0*	**3.8**	*0.1*	**1.2**	*0.2*	**1.2**	*0.7*	**1.2**	*1.1*	**1.2**	*1.4*	**1.8**	*2.1*
Mental retardation		**20.9**	*4.7*	**52.1**	*0.4*	**9.5**	*0.7*	**4.6**	*1.0*	**6.0**	*1.5*	**8.8**	*2.0*	**14.0**	*3.0*	**22.3**	*4.3*	**24.7**	*5.8*	**36.1**	*10.4*
Problems beginning during childhood		**3.1**	*1.2*	**3.4**	*0.2*	**2.8**	*1.1*	**1.6**	*2.0*	**1.5**	*2.1*	**2.4**	*2.3*	**4.1**	*1.0*	**8.9**	*1.0*	**12.5**	*1.0*	**12.0**	*0.6*
Neurological or degenerative illnesses		**5.3**	*5.7*	**8.2**	*0.8*	**8.6**	*3.3*	**4.6**	*2.7*	**3.6**	*2.2*	**4.5**	*3.1*	**3.7**	*3.0*	**3.9**	*3.9*	**4.1**	*5.1*	**8.0**	*14.3*
Epilepsy		**9.1**	*4.2*	**6.7**	*0.4*	**10.5**	*2.9*	**5.5**	*2.2*	**4.2**	*1.6*	**6.8**	*2.8*	**5.6**	*2.5*	**6.9**	*3.0*	**7.5**	*3.7*	**16.5**	*9.8*
Dementia (including Alzheimer’s disease)		**28.7**	*1.2*	**0.0**	*0.0*	**111.2**	*0.0*	**31.1**	*0.0*	**0.0**	*0.0*	**14.3**	*0.0*	**2.4**	*0.0*	**4.5**	*0.0*	**39.0**	*1.0*	**35.0**	*4.8*
Paraplegia		**2.0**	*0.2*	**0.0**	*0.0*	**7.2**	*0.1*	**7.0**	*0.3*	**6.2**	*0.3*	**3.3**	*0.2*	**1.9**	*0.2*	**1.8**	*0.2*	**1.6**	*0.3*	**1.6**	*0.4*
Parkinson’s disease		**2.4**	*0.2*	**0.0**	*0.0*	**0.0**	*0.0*	**0.0**	*0.0*	**0.0**	*0.0*	**0.0**	*0.0*	**3.3**	*0.0*	**2.5**	*0.0*	**2.4**	*0.1*	**2.8**	*0.6*
Cardioneurovascular diseases		**3.7**	*9.5*	**60.7**	*22.9*	**56.8**	*22.7*	**45.0**	*14.1*	**31.0**	*9.3*	**20.3**	*8.3*	**14.1**	*7.2*	**8.6**	*7.3*	**3.0**	*6.7*	**1.2**	*9.2*
Other cardiovascular conditions		**20.1**	*5.5*	**85.7**	*15.1*	**75.8**	*16.9*	**58.6**	*10.9*	**48.7**	*7.5*	**40.5**	*6.9*	**39.1**	*5.0*	**28.3**	*4.0*	**12.2**	*2.9*	**3.0**	*1.7*
Heart rate/conduction problems		**2.3**	*1.3*	**12.4**	*1.4*	**19.9**	*1.3*	**11.0**	*0.5*	**7.9**	*0.6*	**3.3**	*0.5*	**4.7**	*0.9*	**4.1**	*1.1*	**2.8**	*1.4*	**1.6**	*2.6*
Heart failure		**6.7**	*1.2*	**73.3**	*4.3*	**115.4**	*4.9*	**84.4**	*1.3*	**37.4**	*0.4*	**33.6**	*0.5*	**21.7**	*0.5*	**16.2**	*0.9*	**6.2**	*0.9*	**2.6**	*1.5*
Valvular diseases		**5.8**	*0.9*	**34.6**	*0.8*	**56.3**	*1.2*	**33.0**	*0.6*	**25.7**	*0.5*	**8.9**	*0.2*	**24.7**	*0.9*	**12.9**	*0.9*	**6.0**	*0.9*	**3.1**	*1.4*
Sequelae of cerebrovascular accidents (strokes)		**1.7**	*0.8*	**9.8**	*0.4*	**4.2**	*0.3*	**5.2**	*0.3*	**6.1**	*0.3*	**5.4**	*0.3*	**3.1**	*0.3*	**3.6**	*0.7*	**1.6**	*0.8*	**1.3**	*1.7*
Chronic coronary heart disease		**0.7**	*0.7*	**217.0**	*2.3*	**164.2**	*2.1*	**182.1**	*1.4*	**89.7**	*0.6*	**31.6**	*0.2*	**8.0**	*0.2*	**3.5**	*0.4*	**0.5**	*0.4*	**0.3**	*1.0*
Cerebrovascular accident (stroke)		**1.7**	*0.1*	**0.0**	*0.0*	**20.7**	*0.1*	**0.0**	*0.0*	**0.0**	*0.0*	**0.0**	*0.0*	**4.3**	*0.1*	**4.3**	*0.1*	**3.0**	*0.2*	**0.9**	*0.2*
Obliterating arteriopathy of a lower limb		**0.3**	*0.1*	**0.0**	*0.0*	**45.5**	*0.1*	**20.4**	*0.0*	**16.5**	*0.0*	**0.0**	*0.0*	**1.2**	*0.0*	**1.6**	*0.1*	**0.8**	*0.2*	**0.1**	*0.2*
Chronic end-stage renal disease		**1.8**	*0.2*	**0.0**	*0.0*	**0.0**	*0.0*	**7.5**	*0.0*	**6.2**	*0.0*	**11.7**	*0.1*	**5.0**	*0.1*	**3.9**	*0.3*	**1.5**	*0.2*	**1.0**	*0.2*
Hereditary metabolic or amyloid disease		**4.0**	*0.6*	**10.3**	*0.6*	**5.4**	*0.6*	**8.1**	*1.0*	**7.3**	*0.8*	**6.2**	*0.7*	**4.6**	*0.4*	**5.3**	*0.5*	**2.6**	*0.4*	**2.4**	*0.5*
Chronic respiratory illnesses		**1.5**	*6.2*	**5.0**	*8.9*	**3.1**	*23.5*	**2.4**	*12.0*	**1.4**	*5.0*	**1.8**	*4.6*	**1.5**	*3.2*	**1.2**	*3.4*	**1.1**	*4.1*	**0.9**	*5.9*
Diseases of the liver or pancreas		**1.5**	*1.0*	**26.7**	*1.6*	**15.3**	*0.6*	**5.6**	*0.2*	**4.9**	*0.3*	**4.1**	*0.5*	**1.4**	*0.4*	**1.7**	*0.9*	**1.8**	*1.6*	**1.3**	*2.0*
Chronic intestinal inflammatory diseases		**0.6**	*0.2*	**0.0**	*0.0*	**0.0**	*0.0*	**0.0**	*0.0*	**3.5**	*0.2*	**1.2**	*0.2*	**0.6**	*0.2*	**0.4**	*0.2*	**0.9**	*0.5*	**0.4**	*0.3*

*conditions with a frequency ≥ 0.1% are reported.

**Table 3:** Comparison of hospital diagnoses (ICD-10 code)* frequencies for individuals with at least one hospitalization in 2019 by age between individuals with and without DS (rate ratio).

Age (years)		Total (< 65)		< 1		1–4		5–9		10–14		15–19		20–29		30–39		40–49		50–64	
Non DS *N (Million)*	DS N	52.3 RR	35,342 %	0.7 RR	485 %	3.0 RR	2,230 %	4.0 RR	2,903 %	4.0 RR	2,355 %	3.9 RR	2,104 %	7,5 RR	5,773 %	8.2 RR	6,163 %	8.4 RR	6,211 %	12.7 RR	7,118 %
Infectious and parasitic diseases		**3.2**	*0.9*	**2.7**	*3.3*	**3.6**	*5.2*	**4.5**	*1.3*	**3.3**	*0.4*	**4.2**	*0.7*	**2.1**	*0.4*	**2.0**	*0.4*	**2.9**	*0.5*	**3.4**	*0.8*
Viral and other (specified) infections of the intestines		**3.3**	*0.1*	**2.3**	*0.8*	**2.9**	*1.5*	**1.8**	*0.1*	**2.5**	*0.0*	**17.0**	*0.1*	**6.8**	*0.0*	**4.4**	*0.0*	**0.0**	*0.0*	**9.8**	*0.0*
Other infectious and unspecified types of gastroenteritis and colitis		**4.4**	*0.4*	**3.6**	*1.6*	**3.8**	*2.8*	**5.4**	*0.8*	**2.5**	*0.1*	**5.7**	*0.3*	**4.0**	*0.2*	**1.8**	*0.1*	**5.3**	*0.2*	**6.0**	*0.3*
Tumors		**0.5**	*0.5*	**0.0**	*0.0*	**6.3**	*0.7*	**3.5**	*0.3*	**3.3**	*0.3*	**2.4**	*0.4*	**0.8**	*0.2*	**0.7**	*0.4*	**0.5**	*0.6*	**0.4**	*1.0*
Lymphoid leukemia		**10.3**	*0.1*	**0.0**	*0.0*	**27.5**	*0.4*	**15.7**	*0.2*	**27.4**	*0.2*	**20.3**	*0.1*	**14.2**	*0.1*	**5.3**	*0.0*	**0.0**	*0.0*	**0.0**	*0.0*
Myeloid leukemia		**6.1**	*0.0*	**0.0**	*0.0*	**207.6**	*0.3*	**0.0**	*0.0*	**0.0**	*0.0*	**0.0**	*0.0*	**13.1**	*0.0*	**3.4**	*0.0*	**7.0**	*0.0*	**0.0**	*0.0*
Disorders of the blood or the immune system		**1.7**	*0.3*	**12.1**	*1.0*	**5.2**	*0.6*	**4.0**	*0.3*	**3.1**	*0.2*	**2.0**	*0.2*	**2.0**	*0.2*	**1.0**	*0.2*	**1.0**	*0.2*	**1.6**	*0.4*
Iron deficiency anemia		**1.4**	*0.1*	**30.7**	*0.4*	**3.6**	*0.0*	**19.3**	*0.1*	**4.0**	*0.0*	**0.0**	*0.0*	**1.3**	*0.1*	**0.7**	*0.1*	**0.5**	*0.1*	**2.2**	*0.2*
Endocrine, nutritional and metabolic diseases		**1.8**	*0.9*	**29.6**	*4.3*	**9.1**	*1.6*	**2.9**	*0.6*	**4.2**	*1.1*	**3.7**	*1.0*	**2.2**	*0.8*	**1.7**	*0.8*	**0.9**	*0.6*	**1.2**	*0.9*
Hypothyroidism		**71.8**	*0.1*	**102.6**	*1.9*	**178.0**	*0.3*	**0.0**	*0.0*	**121.3**	*0.0*	**0.0**	*0.0*	**76.5**	*0.0*	**0.0**	*0.0*	**35.6**	*0.0*	**26.5**	*0.0*
Type 1 diabetes		**2.8**	*0.2*	**0.0**	*0.0*	**9.5**	*0.2*	**4.3**	*0.2*	**2.4**	*0.3*	**3.0**	*0.3*	**3.4**	*0.3*	**3.0**	*0.2*	**2.0**	*0.2*	**2.0**	*0.1*
Type 2 diabetes		**0.9**	*0.1*		*0.0*	**0.0**	*0.0*	**97.8**	*0.0*	**0.0**	*0.0*	**0.0**	*0.0*	**3.8**	*0.1*	**3.1**	*0.2*	**1.3**	*0.2*	**0.5**	*0.2*
Protein-energy malnutrition		**7.5**	*0.1*	**105.2**	*1.6*	**34.9**	*0.4*	**0.0**	*0.0*	**0.0**	*0.0*	**0.0**	*0.0*	**0.0**	*0.0*	**3.9**	*0.0*	**4.2**	*0.0*	**3.7**	*0.1*
Mental or behavioural problems		**1.2**	*0.4*	**0.0**	*0.0*	**5.0**	*0.4*	**2.4**	*0.4*	**1.1**	*0.3*	**0.6**	*0.3*	**0.5**	*0.2*	**0.8**	*0.2*	**0.4**	*0.2*	**2.5**	*0.9*
Diseases of the nervous system		**3.6**	*2.1*	**10.9**	*1.6*	**24.6**	*4.8*	**16.6**	*3.3*	**11.4**	*2.2*	**8.0**	*1.9*	**3.5**	*1.1*	**2.2**	*1.2*	**1.5**	*1.2*	**3.0**	*3.2*
Epilepsy		**12.1**	*0.9*	**4.0**	*0.2*	**11.7**	*1.3*	**4.5**	*0.4*	**2.3**	*0.2*	**8.2**	*0.6*	**4.9**	*0.3*	**6.2**	*0.4*	**8.7**	*0.6*	**31.5**	*2.6*
Sleep apnea		**8.3**	*1.1*	**66.4**	*1.0*	**107.3**	*3.5*	**57.7**	*2.4*	**62.8**	*1.6*	**47.6**	*1.1*	**17.8**	*0.8*	**6.4**	*0.6*	**3.0**	*0.6*	**2.1**	*0.6*
Ophthalmological diseases		**4.6**	*1.3*	**8.3**	*0.4*	**12.5**	*1.6*	**5.4**	*0.8*	**6.6**	*0.5*	**7.1**	*0.5*	**9.9**	*0.8*	**7.7**	*0.9*	**9.6**	*1.9*	**3.2**	*2.2*
Keratitis		**23.8**	*0.1*	**0.0**	*0.0*	**0.0**	*0.0*	**0.0**	*0.0*	**0.0**	*0.0*	**0.0**	*0.0*	**12.5**	*0.1*	**29.9**	*0.2*	**35.2**	*0.2*	**28.1**	*0.2*
Keratoconus		**30.5**	*0.1*		*0.0*	**1334.8**	*0.0*	**0.0**	*0.0*	**0.0**	*0.0*	**23.5**	*0.2*	**24.0**	*0.2*	**23.9**	*0.2*	**58.6**	*0.2*	**37.2**	*0.1*
Infantile, juvenile or presenile cataract		**4.9**	*0.4*	**0.0**	*0.0*	**65.1**	*0.1*	**23.6**	*0.0*	**91.8**	*0.1*	**57.9**	*0.1*	**70.0**	*0.2*	**28.1**	*0.3*	**15.3**	*0.7*	**3.2**	*1.1*
Other types of cataract (specified or unspecified)		**4.6**	*0.2*	**729.9**	*0.2*	**133.5**	*0.0*	**0.0**	*0.0*	**0.0**	*0.0*	**44.1**	*0.0*	**122.7**	*0.2*	**20.7**	*0.1*	**17.8**	*0.4*	**2.8**	*0.5*
Diseases of the ear and mastoid apophasis		**7.0**	*1.2*	**2.1**	*0.2*	**6.7**	*5.7*	**8.7**	*3.9*	**21.2**	*2.5*	**23.8**	*1.6*	**11.2**	*0.7*	**3.5**	*0.3*	**1.5**	*0.2*	**2.2**	*0.3*
Otitis media		**9.3**	*0.7*	**0.0**	*0.0*	**7.0**	*5.0*	**8.7**	*3.0*	**30.5**	*1.4*	**38.4**	*0.5*	**21.5**	*0.2*	**7.2**	*0.1*	**4.3**	*0.0*	**2.8**	*0.0*
Cholesteatomy of the middle ear		**9.4**	*0.1*		*0.0*	**0.0**	*0.0*	**11.8**	*0.1*	**18.8**	*0.3*	**15.8**	*0.2*	**16.5**	*0.1*	**6.5**	*0.1*	**7.4**	*0.1*	**4.6**	*0.1*
Bilateral conductive deafness		**9.6**	*0.1*	**42.9**	*0.2*	**16.3**	*0.4*	**23.1**	*0.3*	**0.0**	*0.0*	**0.0**	*0.0*	**21.9**	*0.1*	**3.7**	*0.0*	**0.0**	*0.0*	**0.0**	*0.0*
Circulatory diseases		**2.0**	*1.3*	**39.8**	*3.3*	**15.1**	*0.9*	**4.6**	*0.4*	**1.8**	*0.2*	**1.9**	*0.2*	**5.0**	*1.0*	**3.1**	*1.3*	**2.3**	*1.7*	**1.3**	*2.0*
Pulmonary embolism		**5.2**	*0.1*	**0.0**	*0.0*	**0.0**	*0.0*		*0.0*	**0.0**	*0.0*	**0.0**	*0.0*	**1.5**	*0.0*	**5.4**	*0.1*	**4.7**	*0.2*	**6.6**	*0.4*
Pulmonary hypertension		**21.9**	*0.1*	**175.2**	*0.6*	**215.3**	*0.2*	**76.1**	*0.0*	**0.0**	*0.0*	**0.0**	*0.0*	**57.6**	*0.1*	**61.6**	*0.2*	**26.7**	*0.2*	**4.5**	*0.1*
Varicose veins of the lower limbs		**1.9**	*0.2*		*0.0*		*0.0*	**0.0**	*0.0*	**0.0**	*0.0*	**19.7**	*0.1*	**8.6**	*0.3*	**2.9**	*0.4*	**1.7**	*0.4*	**0.8**	*0.2*
Respiratory diseases		**5.2**	*3.7*	**3.1**	*12.4*	**5.1**	*15.6*	**5.4**	*6.8*	**6.1**	*2.3*	**5.3**	*1.8*	**2.0**	*0.8*	**2.9**	*1.2*	**4.5**	*1.8*	**8.9**	*5.1*
Acute rhinopharyngitis or laryngitis		**6.6**	*0.2*	**3.2**	*0.6*	**8.0**	*2.5*	**8.4**	*0.6*	**5.3**	*0.1*	**2.6**	*0.0*	**0.0**	*0.0*	**1.5**	*0.0*	**0.0**	*0.0*	**0.0**	*0.0*
Flu		**7.3**	*0.2*	**0.0**	*0.0*	**5.7**	*0.9*	**7.7**	*0.3*	**10.4**	*0.1*	**14.0**	*0.1*	**4.0**	*0.1*	**4.8**	*0.1*	**1.3**	*0.0*	**14.2**	*0.4*
Pneumonitis		**17.8**	*1.2*	**20.3**	*2.1*	**16.3**	*3.0*	**16.8**	*0.7*	**10.3**	*0.2*	**33.6**	*0.6*	**12.8**	*0.3*	**13.0**	*0.5*	**18.6**	*1.0*	**21.5**	*2.6*
Bronchitis		**14.4**	*0.2*	**11.2**	*0.4*	**9.8**	*0.4*	**13.6**	*0.1*	**12.0**	*0.0*	**8.7**	*0.0*	**10.5**	*0.1*	**23.6**	*0.2*	**10.8**	*0.1*	**17.9**	*0.5*
Bronchiolitis		**6.2**	*0.5*	**3.8**	*10.5*	**7.8**	*4.9*	**53.7**	*0.1*	**0.0**	*0.0*	**0.0**	*0.0*	**0.0**	*0.0*	**0.0**	*0.0*	**15.2**	*0.0*	**7.8**	*0.0*
Tonsil hypertrophy		**5.5**	*0.8*	**0.0**	*0.0*	**5.6**	*5.7*	**4.8**	*3.8*	**9.1**	*1.4*	**7.2**	*0.3*	**1.5**	*0.1*	**0.0**	*0.0*	**1.5**	*0.0*	**3.0**	*0.0*
Asthma		**3.2**	*0.3*	**6.9**	*1.0*	**4.8**	*3.1*	**3.1**	*0.6*	**1.9**	*0.2*	**1.0**	*0.0*	**0.0**	*0.0*	**0.0**	*0.0*	**0.5**	*0.0*	**1.1**	*0.0*
Aspiration pneumonitis		**89.2**	*0.7*	**0.0**	*0.0*	**21.9**	*0.1*	**16.9**	*0.0*	**0.0**	*0.0*	**72.6**	*0.2*	**47.7**	*0.1*	**49.1**	*0.2*	**84.8**	*0.6*	**127.5**	*2.5*
Respiratory insufficiency		**16.9**	*0.4*	**17.7**	*2.5*	**21.7**	*1.2*	**25.2**	*0.2*	**0.0**	*0.0*	**61.1**	*0.3*	**24.2**	*0.1*	**30.7**	*0.2*	**19.8**	*0.3*	**13.3**	*0.7*
Diseases of the digestive system		**1.7**	*4.1*	**2.3**	*2.3*	**4.0**	*2.2*	**5.5**	*3.5*	**3.9**	*4.1*	**1.1**	*4.9*	**1.4**	*3.4*	**1.8**	*3.8*	**1.8**	*4.5*	**1.6**	*5.2*
Problems with the teeth, gums, or jaw		**3.1**	*2.1*	**0.0**	*0.0*	**2.6**	*0.3*	**9.0**	*2.7*	**5.6**	*3.6*	**1.0**	*3.8*	**1.7**	*2.1*	**4.3**	*1.8*	**9.0**	*2.1*	**10.8**	*2.0*
Paralysis of the ileus and intestinal occlusion without hernia		**6.1**	*0.3*	**17.3**	*0.6*	**2.1**	*0.1*	**8.4**	*0.1*	**0.0**	*0.0*	**3.4**	*0.0*	**5.3**	*0.1*	**3.3**	*0.1*	**5.6**	*0.3*	**8.6**	*0.7*
Diseases of the skin and subcutaneous cellular tissues		**1.8**	*0.6*	**3.7**	*0.4*	**4.0**	*0.8*	**3.8**	*0.5*	**2.2**	*0.5*	**1.7**	*1.0*	**1.7**	*0.9*	**1.3**	*0.5*	**1.9**	*0.6*	**1.5**	*0.5*
Diseases of the bones and joints		**0.8**	*0.9*	**6.0**	*0.2*	**2.7**	*0.4*	**2.1**	*0.3*	**3.3**	*0.8*	**1.1**	*0.8*	**0.9**	*0.8*	**0.8**	*0.9*	**0.7**	*1.0*	**0.6**	*1.4*
Diseases of the genitourinary system		**1.2**	*1.2*	**2.2**	*1.9*	**1.4**	*2.3*	**2.5**	*1.6*	**3.7**	*1.1*	**0.3**	*0.2*	**0.5**	*0.4*	**0.6**	*0.7*	**1.0**	*1.1*	**1.7**	*1.9*
Acute tubulointerstitial nephritis		**2.9**	*0.2*	**1.4**	*1.0*	**3.2**	*0.8*	**4.4**	*0.2*	**8.8**	*0.2*	**0.9**	*0.0*	**0.5**	*0.0*	**1.6**	*0.1*	**2.4**	*0.1*	**6.0**	*0.4*
Congenital diseases other than DS		**8.1**	**1.4**	**22.3**	**20.8**	**10.5**	**7.5**	**7.6**	**2.8**	**6.3**	**2.1**	**4.5**	**1.0**	**4.5**	**0.5**	**6.0**	**0.5**	**3.5**	**0.2**	**2.3**	**0.1**
Congenital malformations		**13.5**	*2.3*	**38.9**	*36.3*	**15.5**	*11.1*	**13.3**	*4.9*	**11.6**	*3.8*	**6.9**	*1.5*	**5.5**	*0.6*	**8.8**	*0.7*	**6.2**	*0.4*	**9.6**	*0.5*
Congenital cataract		**52.0**	*0.1*	**47.6**	*0.6*	**45.5**	*0.5*	**50.4**	*0.2*	**121.3**	*0.1*	**0.0**	*0.0*	**0.0**	*0.0*	**0.0**	*0.0*	**104.0**	*0.0*	**118.6**	*0.0*
Hirschsprung disease		**100.6**	*0.1*	**57.2**	*0.8*	**150.2**	*0.4*	**148.1**	*0.1*	**89.4**	*0.0*	**0.0**	*0.0*	**0.0**	*0.0*	**0.0**	*0.0*	**0.0**	*0.0*	**0.0**	*0.0*
Atrial septal defect		**43.4**	*0.8*	**93.1**	*19.4*	**99.0**	*4.7*	**43.5**	*1.0*	**28.3**	*0.6*	**15.8**	*0.3*	**29.9**	*0.3*	**29.7**	*0.3*	**10.1**	*0.1*	**2.8**	*0.0*
Testicular abnormalities or cryptorchidism		**5.9**	*0.3*	**0.0**	*0.0*	**5.7**	*1.9*	**5.9**	*1.1*	**6.9**	*0.8*	**0.0**	*0.0*	**0.0**	*0.0*	**0.0**	*0.0*	**23.3**	*0.0*	**0.0**	*0.0*

*Diagnosis with a frequency ≥ 0.1% were reported.

**Table 4:** Prevalence of LTDs (ICD-10 code)* for individuals with at least one LTD in 2019 by age between individuals with and without DS (rate ratio).

Age (years)		Total < 65		< 1		1–4		5–9		10–14		15–19		20–29		30–39		40–49		50–64	
Non DS *N (Million)*	DS N	52.3 RR	35,342 %	0.7 RR	485 %	3.0 RR	2,230 %	4.0 RR	2,903 %	4.0 RR	2,355 %	3.9 RR	2,104 %	7,5 RR	5,773 %	8.2 RR	6,163 %	8.4 RR	6,211 %	12.7 RR	7,118 %
Infectious and parasitic diseases		**1.2**	*0.4*	**13.5**	*0.2*	**3.3**	*0.2*	**1.8**	*0.2*	**2.5**	*0.2*	**1.1**	*0.2*	**0.7**	*0.2*	**1.5**	*0.2*	**1.2**	*0.2*	**1.2**	*0.2*
Chronic hepatitis B without delta agent		**5.3**	*0.3*		*0.0*	**0.0**	*0.0*	**0.0**	*0.0*	**0.0**	*0.0*	**3.8**	*0.0*	**1.7**	*0.1*	**2.6**	*0.2*	**4.6**	*0.4*	**9.6**	*0.7*
Chronic hepatitis C		**0.8**	*0.1*		*0.0*	**0.0**	*0.0*	**0.0**	*0.0*	**0.0**	*0.0*	**0.0**	*0.0*	**1.6**	*0.0*	**4.6**	*0.2*	**1.4**	*0.1*	**0.3**	*0.1*
Tumors		**0.7**	*1.2*	**29.2**	*0.6*	**13.4**	*0.6*	**8.9**	*0.6*	**6.7**	*0.6*	**5.1**	*0.6*	**2.8**	*0.6*	**1.2**	*0.6*	**0.7**	*0.6*	**0.4**	*0.6*
Malignant testicular tumor		**4.5**	*0.3*	**0.0**	*0.0*	**0.0**	*0.0*	**0.0**	*0.0*	**0.0**	*0.0*	**0.0**	*0.0*	**1.8**	*0.1*	**4.6**	*0.6*	**4.9**	*0.7*	**5.0**	*0.4*
Lymphoid leukaemia		**6.1**	*0.2*	**0.0**	*0.0*	**32.7**	*0.4*	**18.3**	*0.5*	**26.9**	*0.6*	**11.6**	*0.2*	**17.5**	*0.3*	**4.4**	*0.1*	**1.5**	*0.0*	**0.9**	*0.1*
Myeloid leukaemia		**4.6**	*0.1*	**365.0**	*0.2*	**111.2**	*0.3*	**38.3**	*0.2*	**33.1**	*0.2*	**42.1**	*0.3*	**12.2**	*0.1*	**2.6**	*0.0*	**2.1**	*0.1*	**0.5**	*0.0*
Blood disorders		**1.4**	*0.2*	**0.0**	*0.0*	**1.6**	*0.0*	**4.1**	*0.0*	**2.3**	*0.0*	**1.9**	*0.0*	**1.3**	*0.0*	**0.9**	*0.0*	**1.1**	*0.0*	**1.2**	*0.0*
Endocrine disorders		**1.3**	*3.4*	**21.2**	*1.6*	**14.8**	*1.6*	**9.1**	*1.6*	**5.2**	*1.6*	**4.3**	*1.6*	**4.8**	*1.6*	**3.4**	*1.6*	**1.7**	*1.6*	**0.7**	*1.6*
Hypothyroidism		**49.0**	*0.6*	**81.1**	*1.2*	**74.7**	*1.4*	**72.5**	*1.3*	**45.0**	*0.7*	**31.0**	*0.6*	**37.6**	*0.5*	**50.1**	*0.4*	**41.0**	*0.3*	**45.5**	*0.4*
Hyperthyroidism		**11.6**	*0.1*	**0.0**	*0.0*	**121.3**	*0.0*	**201.4**	*0.2*	**15.7**	*0.0*	**53.5**	*0.2*	**17.6**	*0.1*	**20.3**	*0.2*	**9.1**	*0.1*	**3.9**	*0.1*
Type 1 diabetes		**2.2**	*0.8*	**0.0**	*0.0*	**8.6**	*0.3*	**4.4**	*0.4*	**3.3**	*0.7*	**2.8**	*0.7*	**3.3**	*1.0*	**2.5**	*0.9*	**2.0**	*0.9*	**1.6**	*1.2*
Type 2 diabetes		**0.8**	*1.6*	**0.0**	*0.0*	**9.0**	*0.0*	**5.3**	*0.1*	**3.3**	*0.1*	**6.0**	*0.2*	**5.2**	*0.6*	**3.0**	*1.5*	**1.5**	*2.8*	**0.5**	*3.6*
Mental and behavioral problems		**2.9**	*6.8*	**23.5**	*0.6*	**3.4**	*0.6*	**1.9**	*0.6*	**1.8**	*0.6*	**2.7**	*0.6*	**3.3**	*0.6*	**2.8**	*0.6*	**2.5**	*0.6*	**3.5**	*0.6*
Dementia		**26.1**	*0.5*		*0.0*	**148.3**	*0.0*	**41.5**	*0.0*	**0.0**	*0.0*	**0.0**	*0.0*	**0.0**	*0.0*	**2.6**	*0.0*	**40.5**	*0.5*	**32.1**	*2.1*
Mental retardation		**20.8**	*4.2*	**55.1**	*0.4*	**11.7**	*0.6*	**4.7**	*0.8*	**5.3**	*1.1*	**9.8**	*2.0*	**15.4**	*3.4*	**19.9**	*3.5*	**24.0**	*5.0*	**35.7**	*9.3*
Pervasive developmental disorder		**3.3**	*0.6*	**37.4**	*0.2*	**1.2**	*0.2*	**1.3**	*0.8*	**1.5**	*1.0*	**3.4**	*1.4*	**4.2**	*0.7*	**8.4**	*0.6*	**9.1**	*0.5*	**13.2**	*0.4*
Nervous system disorders		**3.1**	*2.6*	**13.7**	*1.0*	**4.6**	*1.0*	**4.3**	*1.0*	**2.7**	*1.0*	**3.2**	*1.0*	**2.7**	*1.0*	**3.4**	*1.0*	**2.6**	*1.0*	**3.5**	*1.0*
Sleep apnea		**10.9**	*0.1*	**365.0**	*0.4*	**190.7**	*0.1*	**65.2**	*0.1*	**66.6**	*0.1*	**90.4**	*0.1*	**195.0**	*0.2*	**64.7**	*0.2*	**8.9**	*0.1*	**3.0**	*0.1*
Ophthalmological diseases		**4.4**	*0.2*	**28.6**	*0.2*	**8.6**	*0.2*	**9.7**	*0.2*	**0.0**	*0.2*	**5.0**	*0.2*	**7.5**	*0.2*	**3.7**	*0.2*	**4.9**	*0.2*	**3.6**	*0.2*
Keratoconus		**26.4**	*0.1*		*0.0*		*0.0*	**0.0**	*0.0*	**0.0**	*0.0*	**0.0**	*0.0*	**21.7**	*0.1*	**16.8**	*0.0*	**26.0**	*0.1*	**59.0**	*0.1*
Diseases of the ear		**4.8**	*0.1*	**0.0**	*0.0*	**17.4**	*0.0*	**14.2**	*0.0*	**6.5**	*0.0*	**0.0**	*0.0*	**7.5**	*0.0*	**4.1**	*0.0*	**4.5**	*0.0*	**1.5**	*0.0*
Diseases of the circulatory system		**1.5**	*2.8*	**46.7**	*3.3*	**41.6**	*3.3*	**44.0**	*3.3*	**21.5**	*3.3*	**9.0**	*3.3*	**11.7**	*3.3*	**5.6**	*3.3*	**1.7**	*3.3*	**0.6**	*3.3*
Diseases of the respiratory system		**2.1**	*0.8*	**0.0**	*0.0*	**8.0**	*0.0*	**5.8**	*0.0*	**5.9**	*0.0*	**2.4**	*0.0*	**3.1**	*0.0*	**2.6**	*0.0*	**1.8**	*0.0*	**0.9**	*0.0*
Asthma		**2.1**	*0.5*	**0.0**	*0.0*	**5.5**	*0.9*	**4.6**	*1.2*	**5.8**	*1.4*	**2.2**	*0.5*	**2.5**	*0.4*	**1.7**	*0.3*	**0.8**	*0.2*	**0.7**	*0.2*
Chronic respiratory insufficiency		**3.1**	*0.2*	**0.0**	*0.0*	**36.2**	*0.3*	**30.2**	*0.2*	**10.0**	*0.1*	**4.2**	*0.0*	**8.9**	*0.1*	**7.7**	*0.2*	**6.9**	*0.4*	**1.4**	*0.3*
Diseases of the digestive system		**0.8**	*0.4*	**24.5**	*0.4*	**7.7**	*0.4*	**12.3**	*0.4*	**2.9**	*0.4*	**1.1**	*0.4*	**0.9**	*0.4*	**0.6**	*0.4*	**1.1**	*0.4*	**0.4**	*0.4*
Diseases of the skin and subcutaneous cellular tissues		**3.0**	*0.1*	**0.0**	*0.0*	**0.0**	*0.0*	**0.0**	*0.0*	**3.4**	*0.0*	**6.2**	*0.0*	**5.5**	*0.0*	**4.4**	*0.0*	**2.3**	*0.0*	**1.5**	*0.0*
Psoriasis		**2.7**	*0.1*		*0.0*	**0.0**	*0.0*	**0.0**	*0.0*	**0.0**	*0.0*	**0.0**	*0.0*	**1.4**	*0.0*	**4.1**	*0.1*	**2.9**	*0.1*	**2.5**	*0.1*
Diseases of the bones and joints		**0.8**	*0.6*	**0.0**	*0.0*	**4.3**	*0.0*	**3.2**	*0.0*	**2.5**	*0.0*	**1.9**	*0.0*	**1.8**	*0.0*	**1.0**	*0.0*	**0.5**	*0.0*	**0.5**	*0.0*
Scoliosis		**2.9**	*0.2*	**0.0**	*0.0*	**6.4**	*0.0*	**4.1**	*0.1*	**2.9**	*0.6*	**1.8**	*0.7*	**3.8**	*0.4*	**4.8**	*0.2*	**4.8**	*0.2*	**2.7**	*0.1*
Disorders of the genitourinary system		**2.0**	*0.3*	**68.7**	*0.8*	**4.9**	*0.8*	**3.0**	*0.8*	**1.6**	*0.8*	**5.9**	*0.8*	**2.9**	*0.8*	**2.7**	*0.8*	**1.9**	*0.8*	**1.3**	*0.8*
Conditions originating in the perinatal period		**4.6**	*0.1*	**4.8**	*1.0*	**5.4**	*1.0*	**3.7**	*1.0*	**1.4**	*1.0*	**2.8**	*1.0*	**2.6**	*1.0*	**0.0**	*1.0*	**10.3**	*1.0*	**0.0**	*1.0*
Congenital malformations		**24.4**	*8.2*	**33.3**	*17.3*	**28.3**	*17.3*	**21.9**	*17.3*	**20.5**	*17.3*	**22.7**	*17.3*	**25.3**	*17.3*	**26.7**	*17.3*	**21.8**	*17.3*	**23.7**	*17.3*
Cardiac malformations		**52.2**	*5.2*	**98.8**	*15.5*	**84.9**	*16.1*	**61.0**	*9.9*	**53.1**	*7.2*	**49.9**	*7.9*	**45.3**	*5.3*	**51.4**	*3.8*	**37.4**	*2.3*	**23.2**	*1.3*
Clubfoot		**4.5**	*0.1*	**5.3**	*0.4*	**4.0**	*0.3*	**2.4**	*0.1*	**3.2**	*0.1*	**3.7**	*0.0*	**9.0**	*0.1*	**0.0**	*0.0*	**13.9**	*0.0*	**13.4**	*0.0*

*Diagnosis with a frequency ≥ 0.1% were reported.

For infectious diseases, IWDS, particularly adults, were more likely to have LTD status for chronic viral hepatitis B without a delta agent (0.3%, RR = 5.3) (Table 4). IDS were also more likely to be hospitalized for intestinal viral infections and other types gastroenteritis and colitis (0.4%, RR = 4.4) particularly during childhood (Table 3).

IDS were more frequently hospitalized (Table 3).Type 1 diabetes was more frequent among the hospital diagnoses (0.2%, RR = 2.8) and LTDs (0.8%, RR = 2.2) of IDS than IWDS (Tables 2, 3). The same was true for hypothyroidism (0.1%, RR = 71.8; 0.6%, RR = 49.0). IDS were more frequently affected by hereditary metabolic diseases or amyloidosis (0.6%, RR = 4.0) (Table 2).

Mental retardation was the most frequent mental or behavioral disorder observed in IWDS (LTD 4.2%, RR = 20.8) (Table 2). The most frequent neurological disorders in IWDS were epilepsy (4.2%, RR = 9.1) and dementia (1.2%, RR = 28.7). IDS were also more likely than IWDS to be hospitalized for sleep apnea (1.1% RR = 8.3, at any age) (Table 3).

IDS were more likely to have ophthalmological diseases (Table 3), such as LTD status for keratoconus, although the frequency of such diseases remained low (0.1%, RR = 26.4). IDS were also more frequently hospitalized for eye diseases (1.3%, RR = 4.6) and ENT conditions (1.2%, RR = 7), such as otitis media, cholesteatoma of the middle ear and conductive deafness.

Cardiovascular diseases were also present in excess among IDS (9.5%, RR = 3.7) (Table 2). An analyses of LTDs showed that there was a very high frequency of cardiac malformations in IWDS (5.2%, RR = 52.2), particularly those under the age of four years (around 15%) (Table 3). Valvular disease (0.9%, RR = 5.8) and heart failure (1.2%, RR = 6.7) were more frequently reported in IWDS, as were rhythm and conduction disorders, albeit to a lesser extent (1.3%, RR = 2.3) (Table 2). The RRs for these conditions were higher in younger patients. Hospitalization for pulmonary hypertension was also more frequent among IDS than among those without DS (0.1%, RR = 21.9), particularly for younger age groups.

Chronic respiratory diseases were more frequent in IDS (6.2%, RR = 1.5) (Table 2), who were also more likely to have LTDs such as asthma (0.5%, RR = 2.1) and chronic respiratory insufficiency (0.2%, RR = 3.1) (Table 3). The most frequent hospital diagnosis in IDS was pneumonitis (1.2%, RR = 17.8), particularly pneumonitis caused by the aspiration of food and other substances (0.7%, RR = 89.2). Tonsillar hypertrophy was also particularly frequent in IWDS under the age of 20 years (0.8%, RR = 5.5). They were also more often frequently hospitalized for bronchitis (0.2%, RR = 14.4), bronchiolitis (0.5% RR = 6.2) and respiratory failure (0.4%, RR = 16.9). IDS were more likely to have LTD status for scoliosis (0.2% RR = 2.9), but were less likely to have diseases of the digestive system (0.4%, RR = 0.8) (Table 2). However, during the first few years of life, they more frequently had LTD status for certain relatively rare diseases or recorded hospitalizations for paralytic ileus and intestinal occlusions without hernia (0.3% RR = 6.1) (Table 3).

LTD status for conditions of the genitourinary system (0.3% RR = 2) was particularly frequent in IDS during the first year of life (0.8%, RR = 68.7) (Table 2). They were also more frequently hospitalized for these diseases (1.2% RR = 1.2), particularly those affecting urethral structures (0.1% RR = 11.3) (Table 3).

### Healthcare use

In 2019, IDS were more likely than IWDS to have at least one visit to any type of healthcare (Table 5). For general practitioners (GP), the frequency of visits was similar between the two groups (88.1%, RR = 1). However, the median number of annual consultations was higher for IDS (5 vs. 3). They also visit more frequently medical specialists and more particularly ENT specialists (22.1%, RR = 3.0), cardiologists (17.2%, RR = 2.6), and neurologists (5.1%, RR = 2.4). They also had a higher frequency of visits for some surgical specialties, such as pediatric (2.1%, RR = 3.1) and thoracic (0.3%, RR = 1.9) surgery. IDS also consulted physiotherapists (17.6%, RR = 1.2) and nurses (45%, 1.7) more frequently than IWDS, particularly for the younger age groups, but they did not consult dentists more frequently (38%, RR = 0.9). The median number of annual visits to healthcare professionals as outpatients was generally similar between the two groups, except for physiotherapists (24 vs. 12) and psychiatrists (4 vs. 3), who were more frequently consulted by IDS.

**Table 5:** Frequency of individuals with DS with at least one outpatient or inpatient visit, emergency department visit or hospital stay in 2019 relative to individuals without DS, by age, and the median number of visits and hospital admissions.

	At least one visit < 65 years old		Rate ratio for at least one visit									Population < 65 years old			
			Age												
	%	Total RR	< 1 RR	1–4 RR	5–9 RR	10–14 RR	15–19 RR	20–29 RR	30–39 RR	40–49 RR	50–64 RR	DS		Without DS	
												Med	IQR	Med	IQR
Physician*															
ENT specialist	**22.1**	**3.0**	5.8	4.7	3.8	5.2	4.0	3.2	2.7	2.3	1.7	1	1–2	1	1–2
Cardiologist	**17.2**	**2.6**	15.7	19.5	11.9	7.3	5.2	6.0	4.3	2.5	1.2	1	1–2	1	1–2
Neurologist	**5.1**	**2.4**	3.3	8.5	4.7	4.0	2.5	2.8	1.9	2.0	2.6	1	1–2	1	1–2
Pediatrician	**15.1**	**2.0**	1.4	1.6	2.1	2.6	3.9	2.1	0.9	2.5	2.5	2	1–4	2	1–4
Endocrinologist	**4.4**	**2.0**	25.4	21.7	11.3	6.9	5.0	3.8	2.5	1.8	0.8	1	1–2	1	1–2
Stomatologist	**2.0**	**2.0**	8.1	15.5	5.9	3.4	1.2	1.2	1.6	2.0	1.5	1	1–2	1	1–2
Nephrologist	**0.8**	**2.0**	7.4	5.3	8.7	9.6	2.7	3.8	2.7	2.1	1.4	1	1–2	1	1–3
Psychiatrist	**5.3**	**1.7**	0.0	1.7	1.1	1.4	1.4	2.1	2.1	1.6	1.6	3	1–7	4	1–10
Pulmonologist	**4.6**	**1.6**	11.5	4.1	1.7	1.1	1.8	2.9	2.6	2.0	0.9	1	1–2	1	1–2
Internal medicine specialist	**3.3**	**1.5**	3.4	2.9	2.7	2.0	2.0	1.2	1.2	1.4	1.2	1	1–2	1	1–2
Ophthalmologist	**37.2**	**1.4**	8.4	4.2	1.7	1.6	1.6	1.6	1.5	1.1	0.9	1	1–2	1	1–1
Rehabilitation doctor	**1.0**	**1.3**	9.3	8.4	6.9	2.0	1.7	1.2	1.1	0.8	1.0	1	1–1	1	1–2
General practitioner	**88.1**	**1.0**	0.8	1.0	1.0	1.0	1.0	1.1	1.1	1.0	1.0	5	2–8	3	2–6
Dermatologist	**9.3**	**1.0**	0.4	0.8	0.8	0.9	1.0	1.3	1.1	1.0	0.7	1	1–2	1	1–2
Gastroenterologist	**4.4**	**1.0**	4.3	2.8	4.0	2.5	1.7	1.3	1.1	1.1	0.8	1	1–2	2	1–2
Gynecologist (total population)	**6.6**	**0.4**	3.6	9.4	8.2	2.6	0.4	0.4	0.4	0.5	0.3	1	1–2	1	1–2
Gynecologist (women only)	**13.3**	**0.5**	4.7	9.3	9.1	2.2	0.4	0.4	0.5	0.5	0.3	1	1–2	1	1–2
Surgeons															
Pediatric	**2.1**	**3.1**	2.5	3.1	3.5	3.0	2.8	6.4	1.2	0.0	0.0	1	1–2	1	1–2
Thoracic and vascular	**0.3**	**1.9**	61.1	108.9	48.3	14.6	3.5	1.9	2.8	1.6	0.6	1	1–2	1	1–2
General	**2.9**	**1.2**	4.9	2.7	2.9	2.0	1.4	1.2	0.9	1.0	0.9	1	1–2	1	1–2
Digestive	**1.2**	**1.2**	6.1	5.5	6.7	4.7	2.0	1.0	0.9	0.6	0.8	1	1–2	1	1–3
Urologic	**1.9**	**1.1**	3.5	1.0	1.7	2.0	1.0	0.7	1.1	1.3	1.1	1	1–2	1	1–2
Orthopedic and trauma	**4.0**	**0.8**	1.6	2.1	1.7	1.3	0.8	0.8	0.7	0.7	0.6	1	1–2	2	1–3
Nurse	**45.0**	**1.7**	7.5	5.8	4.5	3.6	1.6	1.4	1.6	1.7	1.2	2	1–7	2	1–4
Physiotherapist	**17.6**	**1.2**	2.6	7.0	7.3	1.4	1.1	1.1	0.9	0.9	1.0	24	10–46	12	6–23
Dentist	**38.0**	**0.9**	44.6	1.5	0.9	0.9	1.0	1.0	0.9	0.9	0.6	1	1–2	2	1–3
At least one ED visit	**9.1**	**2.4**	3.0	3.8	3.4	2.0	1.7	0.9	1.1	2.4	4.1	1	1–2	1	1–1
Hospital stays															
SSH	**24.2**	**1.6**	1.1	4.2	4.9	4.1	1.7	1.1	1.1	1.4	1.6	1	1–2	1	1–1
Rehabilitation care	**1.5**	**2.3**	37.5	18.1	7.7	4.4	3.0	2.1	1.9	1.7	2.0	1	1–2	1	1–2
Psychiatric hospital	**1.1**	**1.6**	0.0	2.1	1.8	2.5	1.1	1.8	1.9	1.4	1.4	1	1–2	1	1–2
Home hospitalization	**0.6**	**6.0**	23.0	29.5	8.7	3.1	1.3	0.6	0.6	4.9	7.3	1	1–2	1	1–2

*Children could be counted in both categories if they had more than one stay or visit.

**Figure 2:**
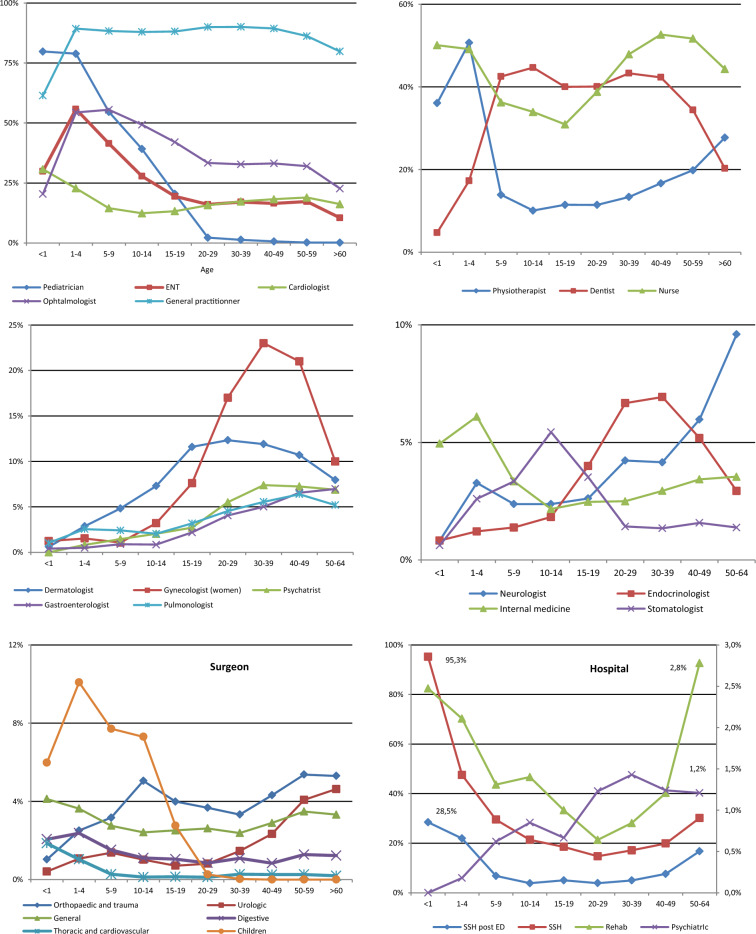
Frequency of at least one visit or admission in 2019 for individual with Down’s syndrome by age.

For some of the most frequently used medical specialties, the frequency of visits was high for the youngest age groups, subsequently decreasing or stabilizing with age (Fig. 2). This pattern was observed for pediatricians, but also for ENT specialists, ophthalmologists, cardiologists and physiotherapists. For other specialists, such as neurologists, gynecologists and dermatologists, the frequency of visits increased with age or stabilized at a uniform level.

IDS also had a greater recourse to emergency services (9.1%, RR = 2.4), particularly for younger age groups, and had higher rates of short stay hospitalisation (24.3%, RR = 1.6), and home hospitalization (0.6%, RR = 6), particularly for younger individuals and those nearing the end of life. A similar, but less marked, pattern was observed for rehabilitation care (1.5%, RR = 2.3) and stays in psychiatric hospitals (1.1%, RR = 1.6) (Table 5, Fig. 2).

## Discussion

This first large nationwide observational study comparing individuals under the age of 65 years with and without DS identified on the basis of hospital diagnoses and LTD status. Individuals with DS more were found to be more likely to live in deprived areas, and to have excess mortality and various comorbidities, particularly for the youngest age groups. They were also more likely to have had at least one stay in a hospital of some type during the course of the year, with various diagnoses, and to have attended outpatient visits with various specialists other than dentists and gynecologists.

The number of IDS of all ages in France was 36,500 in 2019 (0.07%). A study of European countries (2011–2015), based on birth registry data and modeming techniques, including advances in surgery, yielded very similar estimates for the number (35,684) and prevalence (0.07%) of livebirths of IDS in 2015 for France^8^. Global estimates (1900–2015) including elective terminations reported a steady increase in the prevalence of DS after the 80s, with a stead decrease in prevalence once elective terminations were excluded from the calculation, except in the US, which displayed a slight increase even after the exclusion of elective terminations^8^.

The methods used to screen for DS in France were recommended by French National Authority for Health and the regulated by legal decrees published and updated successively in 1997, 2009, and 2018). Physicians inform all pregnant women that nuchal translucency can be measured at the first ultrasound examination, between 11 and 13 weeks ± 6 days, and both physicians and midwives propose blood tests for DS. Organized screening was introduced in the middle of the 1990s. The results obtained, including those for maternal serum markers, were used to calculate the risk of DS during the first or second trimester of pregnancy. Women for whom this screening revealed a high risk of DS were offered a diagnostic examination—amniocentesis or chorionic villus sampling—to determine fetal karyotype. At the end of 2017, the HAS introduced first-trimester screening of maternal blood for free fetal DNA into its strategy for detecting DS in the fetuses of women at high risk.

We observed no decrease in the prevalence of livebirths affected by DS after the 1980s in this study. Instead, there was a slight increase from the early 1990s to 2000, with the implementation of organized screening beginning in France in the middle of the 1990s^27^. Surprisingly, the introduction of screening was followed by a slight increase in prevalence, followed by a stabilization among children aged 0–4 years in 2019. Many possible explanations have been proposed for the variations observed (sociodemographic factors, abortion policies or funded screening and increases in survival, not considered here). Testing for free fetal DNA in maternal blood was officially recommended in 2018 but was introduced the year before, leading to the plateau in prevalence (0.07%) observed for children under the age of four years^27^.

IDS were more likely to be covered by universal health insurance, suggesting a low household income. This pattern was particularly marked for the youngest (< 1 year and 1–4 years old) IDS and their households. Similarly, poor social conditions were found to be more frequent in IDS over the age of 18 years in Denmark^30^. These results may reflect poor access to prenatal healthcare use, a poor understanding of the nature and goals of screening or poor access to specific elective termination procedures subject to specific limitations or requiring a clear understanding and informed consent. These hypotheses are supported by results from an English study (1998–2007) reporting lower rates of antenatal DS detection and elective abortions for DS in more deprived areas^31^.

Moreover, data from representative perinatal surveys performed on the French mainland in 2010 and 2016, indicate that 10% of the women surveyed underwent no DS testing. Testing was not offered to 12% of these women, and 49% refused testing^32^. Women born outside France were less likely to undergo blood tests, because they more frequently refused such testing, even if their antenatal care was adequate. Livebirths affected by DS are more frequent in FOT, where deprivation is more frequent^33^ than on the mainland, particularly for the youngest age groups. FOT also have specific cultural characteristics, higher rates of migration and refusal of testing, even in situations in which perinatal care is adequate, and there are differences between FOT.

It was also reported that, in 2016, for all children under the age of 18 years in mainland France, those in deprived areas, regardless of their DS status and based on the same markers, were more likely to have LTDs, poorer access to specialists and larger numbers of emergency department visits and hospital admissions^34^. Most of the diseases more frequent among IDS identified here have already been described as more frequent in this population in disease-specific studies, based on frequencies or reviews, but rarely in a single study, by age, with a large panel of diseases. Care consumption is also little described. Some of these diseases, such as congenital heart disease in children, have also been implicated in the excess mortality reported for young IDS. Despite improvements in care, excess mortality persists among IDS and is still associated with the severity of various congenital heart defects and extracardiac malformations or complications following treatment for congenital heart diseases, such as pneumonia and respiratory infection or failure^11,35–37^. Nevertheless, in our analysis of LTDs revealed a relatively high frequency of cardiac malformations, particularly in those under four years of age (around 15%), requiring surgical treatment. Coronary artery disease also occurred in adults with DS, but at rates similar to those in the general population, and at a similar age; similar results were obtained for stroke^15^.

Hospitalization for pulmonary hypertension was also more frequent among IDS, and at younger ages. Surveillance of this condition during childhood is important, because it is often associated with cardiovascular diseases; valvular disease, heart failure, rhythm problems and conduction disorders also reported, but to a lesser extent^37^. Chronic respiratory diseases were more frequent in IDS, who were also more likely to have asthma and chronic respiratory insufficiency. Pneumonitis was the respiratory condition most frequently diagnosed in IDS at the hospital, due particularly to the aspiration of food and other substances, as a result of the dysphagia and swallowing disorders frequently occurring in this population^38^. Young people with DS were also more frequently hospitalized for iron deficiency anemia, and for protein-energy malnutrition before the age of four years. They also more frequently had LTD status for certain rare diseases and hospitalizations for paralytic ileus and intestinal occlusions without hernia.

In a study in the USA between 2013 and 17, heart disease, Alzheimer’s disease and other forms of dementia, and cancer were more common causes of death in the youngest adults with DS than in the general population of same age^14^. A similar pattern was observed here. Pneumonia, pneumonitis, and respiratory failure were more frequent in IDS of all ages, with ethnic differences in the cause of death reported for adults with DS in the American study. The risk of death was higher in IDS for most of diseases, but was lower for heart disease in adults, and death rates from cerebrovascular disease were similar to IWDS^5^. Cancers were less frequently recorded as a cause of death in IDS, but this is unsurprising given that IDS tend to die at younger ages. The exception to the rule was some types of leukemia and testicular cancers that occur in young adults. A higher frequency of hospital admissions, for testicular abnormalities or cryptorchidism, was recognized as a risk factor.

We report here that IDS, particularly the youngest, were more frequently admitted to hospital, particularly those with cardiac diseases^21,23^. The frequency of diagnoses differed between the ISD and NSD. Those with DS were more frequently hospitalized at home before the age of 10 years and when nearing the end of life than NIDS. IDS visited specialists and surgeons more frequently, in relation to the conditions more frequent in this population, particularly at young ages. They also visited physiotherapists more frequently.

### Strengths and limitations

This cross-sectional observational study concerned 52.3 million individuals under the age of 65 years covered by the national health insurance system and receiving at least one reimbursement for healthcare in 2019 (98.5% of population).The number of IDS is very similar to that in a study in which the size of this population was estimated, because, as reported here, IDS had high rates of healthcare use, hospital diagnoses and potentially life-threatening or disabling LTDs requiring regular, costly, long-term care.

C2S is allocated for at least one year, with an entitlement to 100% reimbursement for all healthcare expenditure, regardless of the health status of the individuals covered, according to a fixed threshold for household or individual income. It can be renewed annually, subject to the claimant making an application.

## Conclusion

This study is one of the largest and most recent to investigate the prevalence of comorbid conditions in individuals with DS, and is therefore ideal for analyses of certain relatively rare conditions. Our results were obtained in a context in which there neither screening nor access to elective abortion for DS is limited and in which universal healthcare cover is available. They suggest that there is a high frequency of comorbid conditions among IDS born alive. The use of specialist healthcare by these individuals is consistent with improvements in their lifespan and healthcare for the various conditions they present. Nevertheless, excess mortality persists, especially among the youngest. Mothers from deprived areas were more likely to have children with DS, raising questions about recommendations for early screening and the care of IDS, which may be inadequate in deprived areas. These data suggest that improvements are required in comprehensive counselling, prenatal healthcare and, after birth, in social support for all IDS and their families.

## Data Availability

All SNDS data are pseudonymised and individually linkable. The sharing of these data is expressly forbidden by law (sensitive individual data). Access to data is provided subject to prior training and authorization and must be approved by the French independent data protection authority (CNIL, “*Commission Nationale Informatique et Libertés*”). Data are available after request from the Health Data Hub (HDH contact via hdh@health-datahub.fr) for researchers (public or private) meeting the criteria for access to confidential data. Thus, data that support the findings of this study are publicly available after specific request and authorization^25^.

## Abbreviations

SSH: Short stay hospitalisation
ED: Emergency department
Rehab: Rehabilitation
